# BRAVEHeart: a randomised trial comparing the accuracy of Breathe Well and RPM for deep inspiration breath hold breast cancer radiotherapy

**DOI:** 10.1186/s13063-023-07072-y

**Published:** 2023-02-22

**Authors:** Hilary L. Byrne, Elisabeth Steiner, Jeremy Booth, Gillian Lamoury, Marita Morgia, Kylie Richardson, Leigh Ambrose, Kuldeep Makhija, Cameron Stanton, Benjamin Zwan, Regina Bromley, John Atyeo, Shona Silvester, Natalie Plant, Paul Keall

**Affiliations:** 1 ACRF Image X Institute, School of Health Sciences, The University of Sydney, Sydney, Australia; 2 LK Wiener Neustadt, Wiener Neustadt, Austria; 3 Northern Sydney Cancer Centre, Royal North Shore Hospital, Sydney, Australia; 4 School of Physics, The University of Sydney, Sydney, Australia

**Keywords:** Breast cancer, Radiotherapy, Deep inspiration breath hold, Motion monitoring, Visual feedback, Surface monitoring

## Abstract

**Background:**

Deep inspiration breath hold (DIBH) reduces radiotherapy cardiac dose for left-sided breast cancer patients. The primary aim of the BRAVEHeart (Breast Radiotherapy Audio Visual Enhancement for sparing the Heart) trial is to assess the accuracy and usability of a novel device, Breathe Well, for DIBH guidance for left-sided breast cancer patients. Breathe Well will be compared to an adapted widely available monitoring system, the Real-time Position Management system (RPM).

**Methods:**

BRAVEHeart is a single institution prospective randomised trial of two DIBH devices. BRAVEHeart will assess the DIBH accuracy for Breathe Well and RPM during left-sided breast cancer radiotherapy. After informed consent has been obtained, 40 patients will be randomised into two equal groups, the experimental arm (Breathe Well) and the control arm (RPM with in-house modification of an added patient screen). The primary hypothesis of BRAVEHeart is that the accuracy of Breathe Well in maintaining the position of the chest during DIBH is superior to the RPM system. Accuracy will be measured by comparing chest wall motion extracted from images acquired of the treatment field during breast radiotherapy for patients treated using the Breathe Well system and those using the RPM system.

**Discussion:**

The Breathe Well device uses a depth camera to monitor the chest surface while the RPM system monitors a block on the patient’s abdomen. The hypothesis of this trial is that the chest surface is a better surrogate for the internal chest wall motion used as a measure of treatment accuracy. The Breathe Well device aims to deliver an easy-to-use implementation of surface monitoring. The findings from the study will help inform the technology choice for other centres performing DIBH.

**Trial registration:**

ClinicalTrials.govNCT02881203. Registered on 26 August 2016.

**Supplementary Information:**

The online version contains supplementary material available at 10.1186/s13063-023-07072-y.

## Administrative information

Note: the numbers in curly brackets in this protocol refer to SPIRIT checklist item numbers. The order of the items has been modified to group similar items (see http://www.equator-network.org/reporting-guidelines/spirit-2013-statement-defining-standard-protocol-items-for-clinical-trials/).

**Table:** 

Title {1}	BRAVEHeart: A randomised trial comparing the accuracy of Breathe Well and RPM for deep inspiration breath hold breast cancer radiotherapy
Trial registration {2a and 2b}.	This trial was registered on ClinicalTrials.gov on 26 August 2016. The identifier is: NCT02881203. https://clinicaltrials.gov/ct2/show/NCT02881203
Protocol version {3}	v3.0, 13^th^ August 2020
Funding {4}	The BRAVEHeart trial is funded by the National Health and Medical Research Council (NHMRC) Development Grant (APP1073772) and the National Breast Cancer Foundation of Australia Pilot Study Grant (PS-17-055).
Author details {5a}	Hilary Byrne^a^, Elisabeth Steiner^b^, Jeremy Booth^c,d^, Gillian Lamoury^c^, Marita Morgia^c^, Kylie Richardson^c^, Leigh Ambrose^c^, Kuldeep Makhija^a^, Cameron Stanton^c^, Benjamin Zwan^c^, Felix Bockelmann^c^, Regina Bromley^c^, John Atyeo^c^, Shona Silvester^a^, Natalie Plant^a^, Paul Keall^a^ ^a^ ACRF Image X Institute, School of Health Sciences, The University of Sydney, Sydney, Australia ^b^ LK Wiener Neustadt, Austria ^c^ Northern Sydney Cancer Centre, Royal North Shore Hospital, Sydney, Australia ^d^ School of Physics, The University of Sydney, Australia
Name and contact information for the trial sponsor {5b}	The University of Sydney, Sydney, NSW, 2006, Australia
Role of sponsor {5c}	The study sponsor and funders will not influence study design; collection, management, analysis, and interpretation of data; writing of the report; the decision to submit the report for publication.

## Introduction

### Background and rationale {6a}

Radiotherapy is recommended for 87% of all diagnosed breast cancer patients for disease control [1], but it can lead to late cardiac side effects [2]. Deep inspiration breath hold (DIBH) increases the lung volume and moves the heart outside of the irradiation fields, reducing the heart dose [3–7] and thus reducing the cardiac toxicity during breast cancer radiotherapy [2, 8, 9]. Nissen et al. [7] estimated that DIBH reduces the mean heart dose from 5.2 to 2.7 Gy on average and Darby et al. [2] concluded that the rate of major coronary events increased linearly by 7.4% per Gray of mean heart dose with no threshold. The Darby data outlines that it is important to keep the dose to the heart from radiotherapy, especially for young patients, as low as possible and based on their analysis DIBH reduces the increased rate of major coronary events due to the radiation treatment by 20% [2].

Standardly the patients are guided to the breath hold level with audio prompts from the operating radiation therapists. However, Cervino et al. [10] demonstrated that providing the patients with visual feedback by adding a monitor or goggles in addition to audio guidance has demonstrated improvement in the reproducibility of DIBH by 95% and stability by 80% (and for variations >2 mm by 35% and 15%, respectively). Similar findings have been reported by Damkjær et al. [11].

Several systems are currently commercially available to assist the implementation of DIBH in the clinic. These systems include the real-time position management (RPM) system and real-time gating for scanners (RGSC) system (Varian Medical Systems, Palo Alto, CA), the active breathing coordinator (ABC) (Elekta, Stockholm, Sweden), AlignRT (Vision RT Inc., London, UK), Catalyst HD (C-RAD, Uppsala, Sweden), Exac-Trac (Brainlab, Feldkirchen, Germany), and Identify (Varian Medical Systems, Palo Alto, CA).

RPM and RGSC utilise an infrared camera tracking a marker block, standardly placed on the abdomen between the xiphoid process and the umbilicus. ABC uses a spirometer with a valve that is closed for the breath hold. AlignRT, Catalyst HD, Exac-Trac and Identify use 3D surface imaging, requiring in-room installation and/or do not standardly include a visual feedback feature.

**Fig. 1:**
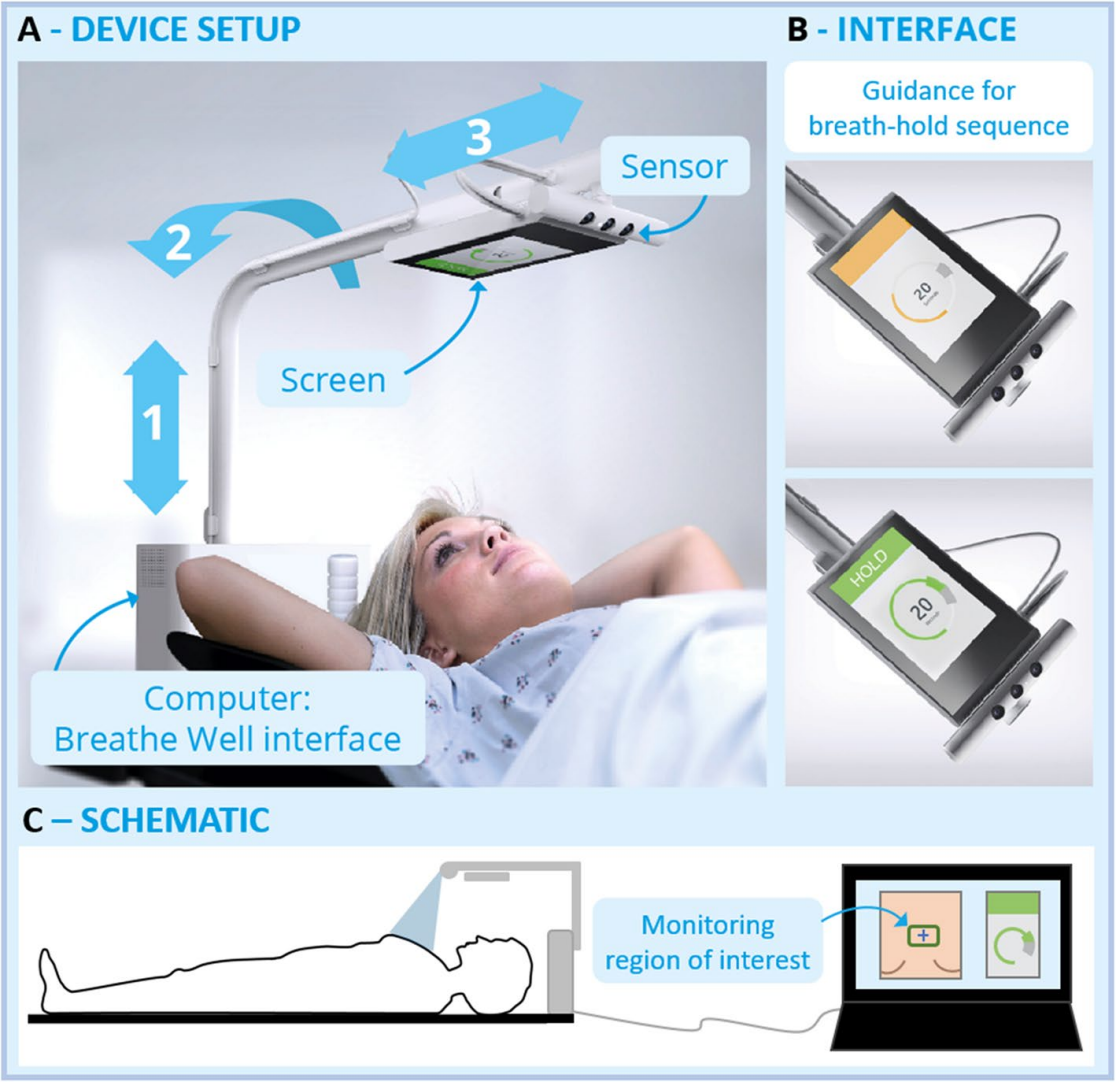
The Breathe Well device. **A** Similar setup on the CT and treatment couch. **B** Patient screen with breath hold position visualisation and instructions. **C** Optical feed and breath-hold depth monitoring central chest area displayed to the radiation therapist

Breathe Well (Fig. 1) is a new audiovisual (AV) feedback device developed at the University of Sydney that may increase the efficacy and improve the workflow of implementing DIBH for breast cancer patients. Breathe Well is clamped on the superior end of the computed tomography (CT) couch or treatment couch and uses depth and optical sensors to directly monitor a user-defined area on a patient’s chest. The positional accuracy and precision of the depth sensor has been shown to be within ± 1.0 mm and the temporal stability of the sensor is sub-mm [12]. The horizontal and vertical axes of the device can be adjusted to fit the patient and immobilisation device dimensions and the device arm can be rotated to enable an unobstructed patient setup and an optimal view of the included screen when the patients are treated with their head tilted (Fig. 1A). The monitoring region of interest is selected by the radiation therapist from within the optical field of view e.g. centred on the sternal tattoo and corresponds to an area of a few square centimetres on the patient’s chest. Within the Breathe Well software application a patient-specific reference breath hold with respect to the normal breathing baseline can be measured and stored. The included screen delivers real-time feedback to the patient regarding their chest position. The breath hold target area (gating window) is highlighted and depending on the workflow status the screen (Fig. 1B) shows an orange wait screen or instructs the patient to hold their breath or relax. An optional breath hold countdown option helps the patient to see how long they still have to hold their breath. At their workstation, the radiation therapists also see the real-time visual feedback indicating when the breath hold depth is acceptable, allowing them to control treatment delivery.

**Fig. 2:**
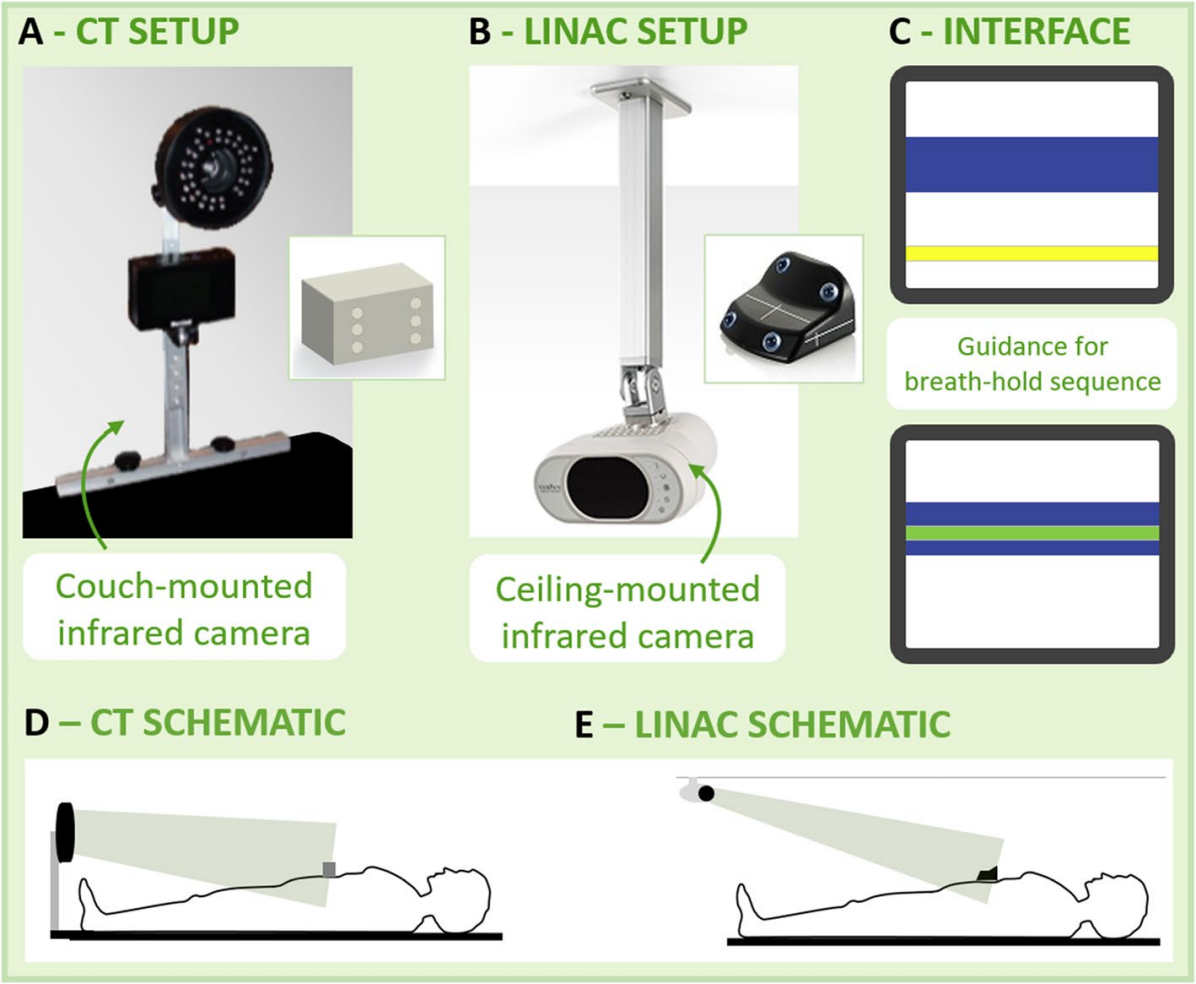
The RPM and RGSC systems. **A** RPM system used at CT. **B** RGSC used during linac treatment. **C** Patient screen with breath hold position visualisation. **D** Camera and marker block setup at CT. **E** Camera and marker block setup at treatment

RPM and RGSC (Fig. 2) similarly have a positional accuracy quoted by the manufacturer of ± 1.0 mm. The RPM system is installed in the CT simulation suite and uses an infrared camera clamped to the foot of the couch while the RGSC system is installed in the treatment suite, with the infrared camera fixed to the ceiling. These infrared cameras monitor a block with reflective dots placed on the patient. For breast DIBH treatment this is commonly placed near the xiphoid process so as to ensure it is not within the treatment field. As with Breathe Well, a patient-specific reference breath hold with respect to the normal breathing baseline can be measured and stored. However, visual feedback is not standardly provided to the patient by these systems, though visualisation of the breathing motion is provided to the radiation therapists. An in-house visual feedback screen has been produced to replicated this real-time breathing visualisation for the patient. In this visualisation, a yellow bar moves up and down to show the anterior-posterior motion. The target gating window is represented as a blue region with the bar changing colour when within the region (Fig. 2C). RPM and RGSC are integrated with the treatment machine to automatically gate the beam.

The primary aim of this study is to test the accuracy and usability of Breathe Well to assist DIBH during breast cancer radiotherapy.

### Objectives {7}

The primary objective of the study is to test the accuracy and usability of Breathe Well to assist DIBH during breast cancer radiotherapy. Breathe Well will be tested against the commercially available real-time position management (RPM) / respiratory gating for scanners (RGSC) system (Varian Medical Systems, Palo Alto, CA), which has been extended in-house to provide a screen for visual feedback.

The primary hypothesis is that the accuracy of Breathe Well in maintaining the position of the chest during DIBH is superior to the RPM/RGSC system. Because Breathe Well monitors the chest directly, while the RPM/RGSC system uses a marker block positioned on the upper abdomen, Breathe Well has the advantage of utilising a more immediate surrogate of the chest wall position. In addition, we hypothesise that Breathe Well is more user-friendly as it was specifically designed for guiding breast cancer patients through DIBH treatments.

Secondary objectives:

1. To measure the setup times for both the Breathe Well and the modified RPM/RGSC system for all fractions. We hypothesise that Breathe Well can be set up faster than the modified RPM/RGSC system.
2. To investigate patient comfort and user friendliness of Breathe Well via a patient survey (Supplementary Materials). We hypothesise that the patient comfort and usability of Breathe Well is superior to the Varian RPM system.
3. To investigate staff perception of Breathe Well via a technology assessment survey (Supplementary Materials). We hypothesise that the staff perception of Breathe Well is superior to the Varian RPM/RGSC system.
4. To develop — offline — the use of the electronic portal imaging device (EPID) for real-time MLC tracking during monitoring the chest wall position in breast radiotherapy.
5. To investigate, using dose reconstruction, the estimated dose delivered during radiotherapy and compare this with the planned dose.

### Trial design {8}

**Fig. 3:**
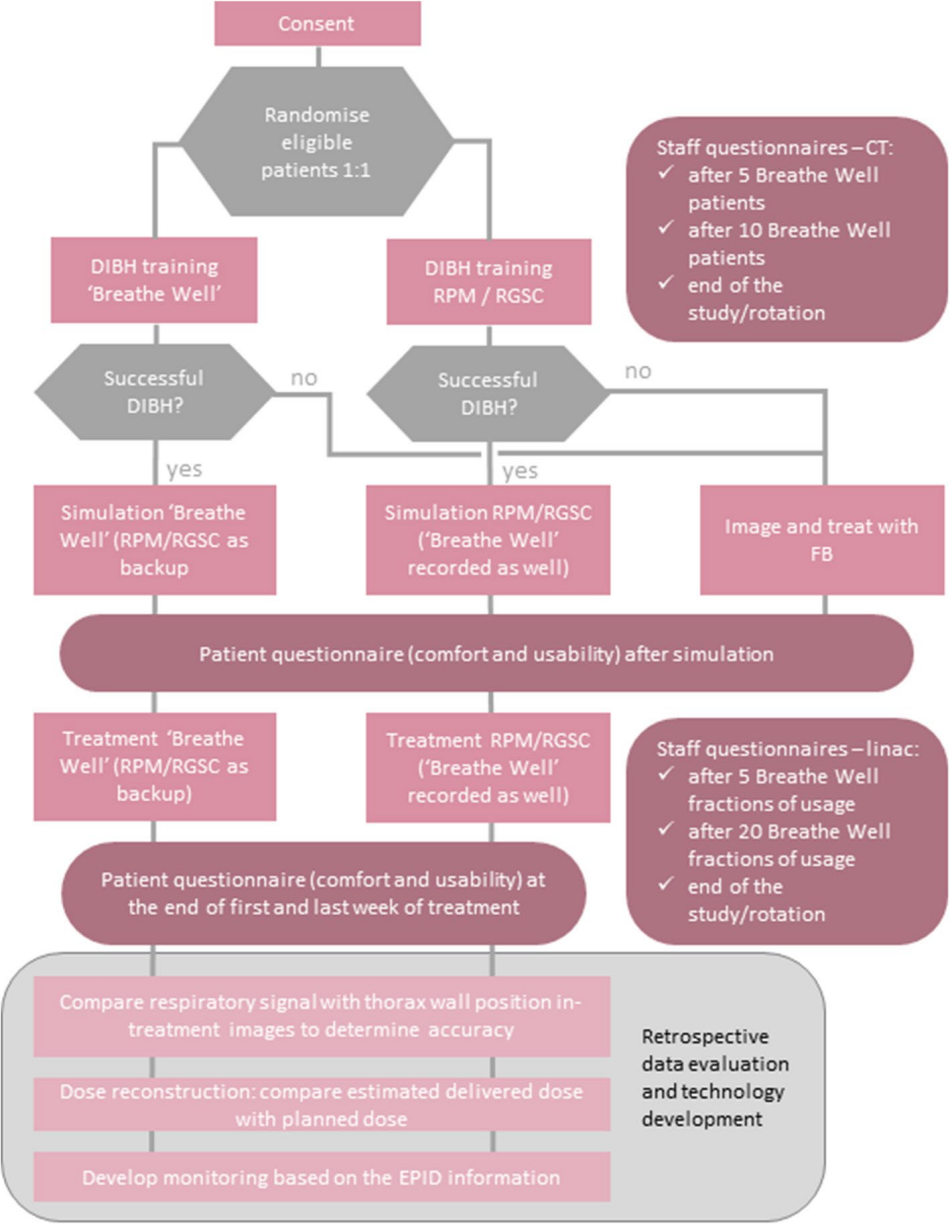
The BRAVEHeart study schema

The trial design is a randomised, prospective superiority trial with an experimental (breathe well) and a control (RPM/RGSC) arm allocation ratio 1:1. Patients receiving radiotherapy for left-sided breast cancer will be randomised into two equal groups. The experimental arm will manage breath holds with audiovisual biofeedback from the Breathe Well device. Patients in the control arm will manage breath holds using an in-house developed audiovisual feedback for the RPM/RGSC system. The trial schema is summarised in Fig. 3.

## Methods: participants, interventions and outcomes

### Study setting {9}

Study participants will be recruited and treated at the Royal North Shore Hospital, St Leonards, NSW, 2065, Australia.

### Eligibility criteria {10}

Inclusion — patients

1. Left-sided breast cancer patients (invasive and in situ)
2. To be treated in the supine position
3. Ability to perform a ≥ 20-s breath hold
4. > 18 years old
5. Female
6. An ECOG score from 0 to 2
7. Able to read and complete questionnaires in English
8. Able to give written informed consent and willingness to participate and comply with the study

Exclusion — patients

1. No regional lymph nodes involved or at risk
2. No pregnant/lactating women

### Who will take informed consent? {26a}

Potential participants will be identified by their consulting clinician who will explain the study, including risks and benefits and give the opportunity for informed discussion. They will receive a copy of the informed consent form to carefully read and take home for further analysis if necessary. Trained clinical trials staff at Royal North Shore Hospital will obtain informed consent from patients prior to their CT simulation appointment.

### Additional consent provisions for collection and use of participant data and biological specimens {26b}

Not applicable.

## Interventions

### Explanation for the choice of comparators {6b}

The choice was made to compare the novel Breathe Well device to RPM/RGSC as an existing widely adopted commercial system in clinical use for breath hold management.

### Intervention description {11a}

Training and simulation: The patients will be trained to perform DIBH with the assigned system and assessed for ability to perform a 20-s breath hold (at 80–90% of the maximal lung capacity). Based on this screening the patient will be deemed suitable for DIBH. For patients suitable for DIBH the breath hold AV guidance while acquiring the planning CT scan will be provided by the assigned system. The unallocated system will be set up to passively record motion. The RPM/RGSC marker block is not within the field of view of the Breathe Well camera, and the Breathe Well device does not interfere with RPM/RGSC tracking the marker block. The reference breath hold depth will be recorded for both systems.

Treatment delivery: For treatment, similarly to simulation, both systems will be set up to monitor breathing motion. The patient will be guided with AV biofeedback from the assigned system depending which arm of the trial they have been randomised to. The unallocated system will passively acquire data but not interfere with treatment in any way. Prior tests showed that no interferences and difficulties are anticipated when operating the ‘Breathe Well’ system and the RPM/RGSC system at the same time. If any treatment fraction cannot be delivered due to a failure of the Breathe Well device, this will be recorded. The treatment will continue at the physician’s discretion either (1) using the RPM system, (2) the fraction will be rescheduled or (3) delivered using free breathing.

### Criteria for discontinuing or modifying allocated interventions {11b}

If the patient is not suitable for DIBH, the standard clinical workflow for free breathing (FB) is followed. The patient will complete the patient experience questionnaire and progress to treatment with free breathing using no breath hold management system and with no further study interventions.

Patients are entitled to withdraw consent at any time and will revert to standard of care treatment with RPM/RGSC. Outcome data for these patients will still be collected.

### Strategies to improve adherence to interventions {11c}

Not applicable. Assessment of ease of use of treatment with the Breathe Well device is the aim of this trial, so non-adherence to the intervention should be recorded. Inability to carry out DIBH will result in free-breathing treatment as per current standard of care.

### Relevant concomitant care permitted or prohibited during the trial {11d}

No restrictions on concomitant care being offered as per standard hospital practice.

### Provisions for post-trial care {30}

Participants will receive post-treatment care as per standard hospital procedure. Compensation could be available through sponsor insurance.

### Outcomes {12}

Primary outcome:

Treatment accuracy: the specific measurement made will be the average chest wall position extracted from the EPID images captured at each treatment fraction. The metric used for analysis will be the ‘displacement’ or difference between this measured position on each captured image frame and the planned average chest wall position from the digitally reconstructed radiograph (DRR). The average per fraction displacements will be compared across the two arms of the trial.

Secondary outcomes:

1. Setup time: The appointment duration will be measured as the time recorded in the ARIA patient management system between patient check-in and appointment end for each treatment fraction, and aggregated for each arm of the trial.
2. Patient comfort and user friendliness. A patient survey (see Supplementary Materials) will be delivered at the timepoints indicated in the “Participant timeline {13}” section and Fig. 3: after simulation, and at the end of the first and last weeks of treatment.
3. Technology assessment: A staff survey (see Supplementary Materials) will be delivered at the timepoints indicated in Fig. 3: after 5 and 10 sessions using Breathe Well at CT simulation and after 5 and 20 sessions using Breathe Well at treatment.
4. To develop — offline — the use of the electronic portal imaging device (EPID) for real-time MLC tracking during monitoring the chest wall position in breast radiotherapy. This work will be reported as future research outputs.
5. Estimated dose delivered. Measured chest wall displacement relative to planned position (taken from the EPID images captured at treatment time, see the primary outcome) will be used to apply an isocentre shift in the treatment planning system, following the approach of Doebrich et al. [13]. Hence the delivered dose will be estimated for each patient during each treatment fraction and aggregated for each arm of the trial.

### Participant timeline {13}

Table 1 shows the schedule of enrolment, interventions, and assessments. Initial toxicity baseline will be taken at enrolment, and again at post-treatment follow-up at 6 weeks and every 6 months for three visits.

**Table 1:**
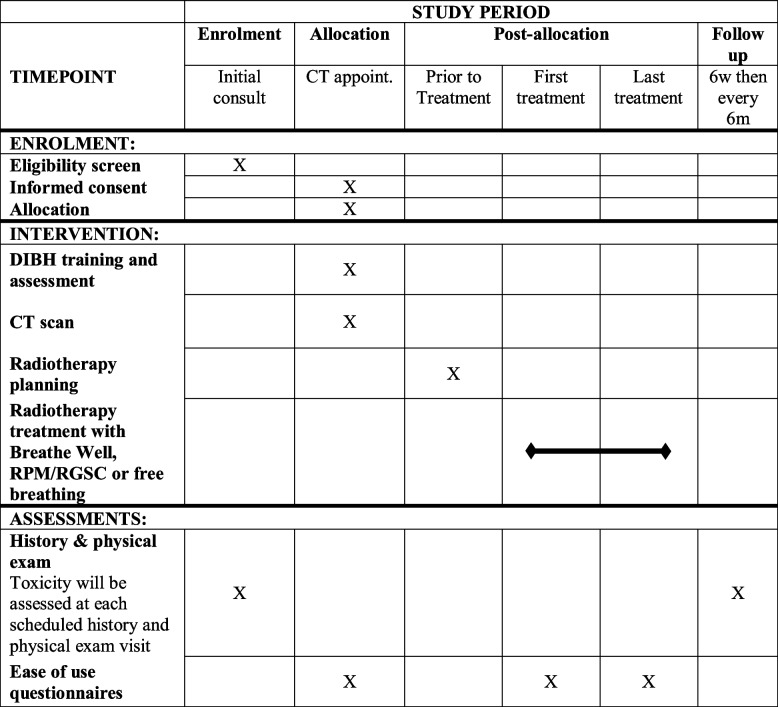
Schedule of enrolment, interventions, and assessments

### Sample size {14}

The sample size was estimated by assuming that the systematic error in treatment accuracy, Σ, will be smaller for patients on the experimental (Breathe Well) arm than the control (RPM) arm. Previously published data for a different surface monitoring system (AlignRT: VisionRT, London, UK) is given by Kanphet et al. [14] who found a systematic error of 0.46 mm in the vertical direction. For comparison, Lutz et al. [15] found a systematic error of 1.33 mm with RPM. Assuming this study will recruit a comparable patient cohort, that any differences in treatment technique will not affect the target motion, and assuming normal distribution of the systematic error in both arms of the trial and independence of the two arms, the F-test for equality of two variances estimates 36 patients will be needed for statistical significance with a power of 0.8 and alpha error probability of 0.05. The sample size for this study was set at 40 to account for an estimated 10% dropout rate for patients who cannot maintain a breath hold and are treated with free breathing.

### Recruitment {15}

Patients suitable for inclusion will be identified by the breast cancer multi-disciplinary team clinicians. Participation in the trial will be offered to the patient by their consulting radiation oncologist and consent will be taken by delegated staff at the hospital.

Recruitment rate is expected to be relatively high due to the non-invasive nature of the intervention proposed, the fact that no extra appointments are required and no extra radiation dose will be delivered, and the number of eligible patients treated at the site.

## Assignment of interventions: allocation

### Sequence generation {16a}

A total of 40 patients will be recruited. After an eligibility assessment and informed consent, participants will be assigned to the experimental arm or control arm with allocation ratio 1:1. The randomisation sequence and grouping will be generated by one researcher who will not be involved directly in patient screening, enrolment or assessment, and uploaded to Research Electronic Data Capture (REDCap) [16, 17]. No stratification will be utilised. The randomisation tables were set up using permuted blocks with random varying block sizes of 4 and 6.

### Concealment mechanism {16b}

The BRAVEHeart trial uses allocation concealment with patients randomly assigned to each treatment arm in REDcap as described above in the “Sequence generation {16a}” section. As the trial is to use one device versus another, the arm a patient is assigned to cannot be concealed to the patient or the treatment team.

### Implementation {16c}

Randomisation is performed at the site electronically by the hospital clinical trials staff using REDCap. The allocation sequence is locked down within the REDCap database and is not accessible to anyone.

## Assignment of interventions: blinding

### Who will be blinded {17a}

No blinding will be used in this trial.

### Procedure for unblinding if needed {17b}

Not applicable.

## Data collection and management

### Plans for assessment and collection of outcomes {18a}

Collection of data during treatment will be initiated by the radiation therapists delivering the treatment. Clinical trials staff will administer the ease-of-use questionnaires (see Additional file 1) with the results entered in REDCap. Clinicians will administer baseline and toxicity questionnaires, with the results entered in REDCap.

### Plans to promote participant retention and complete follow-up {18b}

Participants are only required to attend treatment and follow-up appointments as normally required for their treatment and no dropout is expected. Clinicians and radiotherapy staff will be trained to answer any participant questions or concerns about the trial.

### Data management {19}

Respiratory data (‘Breathe Well’ and RPM/RGSC), CT images, MV frames from during the irradiation, and demographic information will be collected from the subjects. At the randomisation stage of the study, patients will receive a trial ID. The data saved for the trial will be under this de-identified trial ID. A separate key of the subject study number and their medical record number will be securely stored by the chief investigator to allow re-identification if necessary. This master list for re-identification will remain at the Royal North Shore Hospital. Only the principal investigator will have the ability to re-identify subjects. Questionnaire and toxicity information will be entered in REDCap and collated information transferred to the same secure drive at the University of Sydney for storage. The data will be stored for 15 years as per clinical trial guidelines. Data sent from the Northern Sydney Cancer Centre, Royal North Shore Hospital in accordance with the study site’s ethics and security allowances and protocols to the study site University of Sydney will be anonymised but re-identifiable. Patient data could be made re-identifiable to obtain additional clinical information for the data analysis stage of the project, but only by the principal investigator. The de-identified data will be stored at the University of Sydney on a secure, password-protected backed-up database that will be created, much the same as what we have designed for previous University of Sydney studies.

### Confidentiality {27}

At the hospital, copies of questionnaires will be stored in a locked cabinet. Digital information will be stored on the secure local network. Data will be anonymised before leaving the hospital and being transferred to the University of Sydney for storage on a secure, password-protected drive accessible by authorised researchers only. The anonymisation table will remain at Royal North Shore Hospital.

### Plans for collection, laboratory evaluation and storage of biological specimens for genetic or molecular analysis in this trial/future use {33}

Not applicable.

## Statistical methods

### Statistical methods for primary and secondary outcomes {20a}

Primary outcome:

Treatment accuracy: a generalized estimating equation model with exchangeable correlation structure which adjusts for repeated measurements within individuals will be used to test the per fraction average chest wall displacements for all patients on each arm, testing for superiority (smaller chest wall displacement) of the Breathe Well arm compared to the RPM arm. No covariate adjustment, subgroup or sensitivity analysis is planned.

Secondary outcomes:

1. Setup time: Median and range of the appointment time will be reported for each arm. No statistical analysis is planned as clinically significant variation in the appointment time is highly dependent on hospital procedures and workflow.

2. Patient comfort and user friendliness and 3. Technology assessment: The questionnaire tools used for secondary aims 2 and 3 are not validated tools so will only be used for qualitative analysis. The median and range response scores will be reported for each arm for each question.

5. Estimated dose delivered: If the primary outcome shows superiority of the Breathe Well arm in treatment accuracy, the same model will be used to test for superiority (smaller dose differences) of the dose distributions between the Breathe Well arm and the RPM arm. If not, a qualitative analysis will be carried out identifying any patient anatomy- and treatment planning-related points of interest.

### Interim analyses {21b}

Not applicable.

### Methods for additional analyses (e.g. subgroup analyses) {20b}

Not applicable.

### Methods in analysis to handle protocol non-adherence and any statistical methods to handle missing data {20c}

Sample size estimation includes an estimation of patients who will be unable to perform DIBH.

### Plans to give access to the full protocol, participant level-data and statistical code {31c}

Only non-identifiable data may be available for other scientific research, e.g., non-identifiable data placed on a well-controlled university site, upon request. The data sharing platform is a secure online storage solution (“CloudStor”) provided through the University of Sydney. The data will be stored as a password-protected, encrypted Zip-file. In order to download/decompress the data, participating researchers agree to the terms of use for the data, including (i) that the data is not to be published or otherwise redistributed without the express consent of the original investigator(s) and (ii) that the data is forbidden to be used for any commercial purpose.

## Oversight and monitoring

### Composition of the coordinating centre and trial steering committee {5d}

Our steering committee (investigators and sub-investigators including consumer representatives) will meet monthly to monitor the conduct of the study and assess progress. In addition, the chief and majority of sub-investigators will maintain weekly contact via email and face-face or teleconference meetings in order to facilitate implementation of the study and provide quality assurance to all aspects of the study. The chief investigator will be on-site to personally conduct, oversee, and supervise all of the activities

### Composition of the data monitoring committee, its role and reporting structure {21a}

The imaging modalities that are used in this study are approved for clinical practice, therefore this study we will not nominate a separate Data and Safety Monitoring Board.

### Adverse event reporting and harms {22}

The principal investigator and sub-investigators will report adverse events to the Radiation Safety Officer on site and to the Human Research Ethics Committee and the Research Governance Officer within 72 hours of the event occurring unless immediate notification is required.

Radiation therapy has known potential adverse effects. No additional adverse events are expected related to the introduction of new technology in the Breathe Well arm. The following are the known potential adverse events to radiotherapy of the breast: pneumonitis, radiation fibrosis, dyspnoea, dysphagia, odynophagia, pleuritic pain, oesophagitis, fistula, respiratory failure, sleep apnoea, fatigue, stenosis, lung function, nausea, sexual function.

### Frequency and plans for auditing trial conduct {23}

Auditing will only be conducted if required by the funder or Human Research Ethics Committee.

### Plans for communicating important protocol amendments to relevant parties (e.g. trial participants, ethical committees) {25}

Any amendments to the protocol will be reviewed and approved by the Human Research Ethics Committee and communicated to all relevant parties by the lead investigator.

### Dissemination plans {31a}

The results of this study will be published in internationally recognised peer-reviewed journals and presented at international/national conferences. Non-identifiable data may be available for other scientific research on application to the lead investigator.

## Discussion

Successful completion of this trial and positive results of the primary hypothesis will demonstrate that DIBH with AV biofeedback can be achieved using a simple device without the need for an in-room installation. The ease of use of the Breathe Well device should encourage clinical adoption of this technique to maximise the number of patients who can benefit from DIBH. From a patient perspective, access to a system offering biofeedback allows patients to be active participants in their treatments.

In addition to performing DIBH for left-sided breast cancer patients, a possible future pathway is to investigate the potential of Breathe Well for patients treated in the prone position and for right-sided breast cancer patients, where a reduction in lung dose can be achieved [4].

## Trial status

Protocol version number: 3.0 (13 August 2020)

Date recruitment began: 24th October 2018

Approximate date when recruitment will be completed: 30th September 2021

The original trial protocol was amended in August 2020 following an interim analysis of the initial 10 patients, with recruitment recommenced in Jan 2021.

## Supplementary Information

**Additional file 1. **Biofeedback Radiation Therapist Survey. Biofeedback Patient Survey.

## Data Availability

Non-identifiable data from the trial will be available from the corresponding author on reasonable request.

## Abbreviations

AV: Audio-visual
BRAVEHeart: Breast Radiotherapy Audio Visual Enhancement for sparing the Heart
CT: Computed tomography
DIBH: Deep inspiration breath hold
MV: Megavoltage
RPM: Real-time position management system (Varian Medical Systems, Palo Alto, CA)
