# Supplementary material for: BRAVEHeart: a randomised trial comparing the accuracy of Breathe Well and RPM for deep inspiration breath hold breast cancer radiotherapy
Source: Trials. 2023 Feb 22;24:132. doi: 10.1186/s13063-023-07072-y (PMC9945402; doi:10.1186/s13063-023-07072-y)
Supplement: Supplementary file 1 — Additional file 1. Biofeedback Radiation Therapist Survey. Biofeedback Patient Survey. [file 13063_2023_7072_MOESM1_ESM.zip › BRAVEHEART_patient_survey_v2.1_2017_04_13_cleanR2.docx]

**Biofeedback Patient Survey**

**Goal:**

In this study we are testing a novel biofeedback system against an existing one. The system measures your breath hold level and shows it to you on a screen. It should assist you in performing a reproducible and stable deep inspiration breath hold (DIBH). We would like to evaluate your experience with the biofeedback guidance system you are treated with. We rely on your feedback to identify any areas where you feel development is needed to improve the biofeedback experience and greatly appreciate your input.

**Introduction:**

Deep inspiration breath hold has been introduced because it might help to reduce the amount of heart that is covered by the radiation treatment beam. You will be asked to hold your breath while the radiation beam is switched on. We want to know how difficult this is for you. The screen of the biofeedback system should help you to produce consistent breath holds. The biofeedback technology should assist in improving the accuracy of the radiation treatment.

We would like to understand your experience with DIBH and audiovisual guidance so we can make it as easy as possible for future patients.

Today’s date: ________________ (DD/MM/YYYY)

Your age: ________________

**To be completed by the Clinical Research Associate:**

**System:**

**#:** ________________  Breathe Well

RPM

This questionnaire consists of 9 questions. For some questions you are presented with a scale on which you are required to choose between two extremes. For these questions, please place a cross at a point on the line that best summarises your experience (example below).

| Very Easy |  | Very Difficult |
| --- | --- | --- |
|  |  |  |

For other questions you are required to place a cross in a box that best describes your experience, followed by the opportunity to give a reason for your choice. In the space provided after question 9, please provide comments concerning your experience or suggestions for improvement.

| 1. **How easily could you hold your breath for the required time?** |
| --- |

| Without any difficulty |  | Cannot do it at all |
| --- | --- | --- |
|  |  |  |

| 1. **How does the breath-hold experience make you feel?** |
| --- |

| As anxious as I could be |  | As unconcerned as I could be |
| --- | --- | --- |
|  |  |  |

| 1. **Did you have any difficulties seeing the screen?** |
| --- |

Yes  No If yes, why? ………………………………………………………………………………….

1. **Did you have any difficulties hearing the breath hold instructions from the staff?**

Yes  No If yes, why? ………………………………………………………………………………….

1. **How often do you use a computer (e.g.: PC, laptop, smart phone, iPad, tablet)?**

Every day  Most days  Once a week  Once a month  Never

1. **How comfortable did you feel with the breath hold screen? (Position of screen, distance to screen, etc.)**

| Extremely comfortable |  | Not comfortable at all |
| --- | --- | --- |
|  |  |  |

Comments (optional) …………………………………………………………………………………………………………………………

1. **How easy was it for you to follow the visual information on the screen to reach your breath hold level and hold your breath at the correct level?**

| Impossible |  | Very easy |
| --- | --- | --- |
|  |  |  |

Comments (optional) …………………………………………………………………………………………………………………………

1. **How helpful did you find the audio instructions from the radiation therapists to reach your breath hold level and hold your breath at the correct level?**

| Extremely helpful |  | Not helpful at all |
| --- | --- | --- |
|  |  |  |

Comments (optional) …………………………………………………………………………………………………………………………

1. **In the space below, please provide any comments about your experience, or suggestions on how we can improve the biofeedback system.**
