# Supplementary material for: BRAVEHeart: a randomised trial comparing the accuracy of Breathe Well and RPM for deep inspiration breath hold breast cancer radiotherapy
Source: Trials. 2023 Feb 22;24:132. doi: 10.1186/s13063-023-07072-y (PMC9945402; doi:10.1186/s13063-023-07072-y)
Supplement: Supplementary file 1 — Additional file 1. Biofeedback Radiation Therapist Survey. Biofeedback Patient Survey. [file 13063_2023_7072_MOESM1_ESM.zip › BRAVEHEART_staff_survey_v2.2_2017_05_24_cleanR2.docx]

**Biofeedback Radiation Therapist Survey**

**Goal:**

In this study we are testing a novel biofeedback system (Breathe Well) against an existing one (Varian’s RPM). The systems assist the patients to perform a reproducible and stable deep inspiration breath hold (DIBH). We would like to evaluate your experience with the biofeedback guidance systems, their usability and whether you found biofeedback beneficial for the patient. We rely on your feedback to identify any areas where you feel development is needed to improve the biofeedback experience for both the patients and the operators. We would greatly appreciate your input.

This survey is expected to take only 5 minutes to complete; however; participation is entirely voluntary. You will get a participant’s ID when taking part in the survey, because we are collecting data from you at different time points. The investigators at the University of Sydney will not be able to identify you. The key for re-identification will stay with the Clinical Research Associate at RNSH.

**Situation and time for the Breathe Well system:** Please tick the situation and time you are completing this current survey in relation to using the Breathe Well system.

Simulation/CT

after 5 patients

after 10 patients

end of the study or rotation
 Number of patients
 simulated: _____*)

Linear Accelerator

after 5 fractions

after 20 fractions

end of the study or rotation

Number of fractions
 treated: _____*)

*) if you do not know the exact number, please give us an estimate using ~number

Today’s date: ________________ (DD/MM/YYYY)

Your years qualified: ________________

**To be completed by the Clinical Research Associate:**

**Participant’s ID:** ________________

This questionnaire consists of 8 questions. For six of the questions you are presented with a scale on which you are required to choose between extremes. For these questions, please place a cross at a point on the line that best summarises your experience (example below).

| Very Easy |  | Very Difficult |
| --- | --- | --- |
|  |  |  |

For question 6 place a cross in the box that would be your choice of the two DIBH systems, followed by the opportunity to give a reason for your choice. In the space provided after question 8, please provide comments concerning your experience or suggestions for improvement of the two systems.

| 1. **What additional level of confidence reaching the breath hold do patients show using the biofeedback systems (RPM and Breathe Well)?** |
| --- |

| Patients appear a lot more confident |  | Patients appear a lot more stressed |
| --- | --- | --- |
|  |  |  |

| 1. **How easy was the setup of the Breathe Well system?** |
| --- |

| Extremely cumbersome |  | Extremely easy |
| --- | --- | --- |
|  |  |  |

| 1. **How easy was the setup of the RPM system (plus screen)?** |
| --- |

| Extremely cumbersome |  | Extremely easy |
| --- | --- | --- |
|  |  |  |

| 1. **How easy was the Breathe Well system to operate?** |
| --- |

| Extremely easy |  | Extremely cumbersome |
| --- | --- | --- |
|  |  |  |

| 1. **How easy was the RPM system (plus screen) to operate?** |
| --- |

| Extremely easy |  | Extremely cumbersome |
| --- | --- | --- |
|  |  |  |

| 1. **Which of the two systems would you prefer to operate for deep inspiration breath hold treatments?** |
| --- |

Breathe Well  RPM Why? ……………………………………………………………………………….

| 1. **(Only for simulation) Compared to the RPM system, how time efficient did you find the successful coaching of patients with the Breathe Well system?** |
| --- |

| Much faster |  | Much slower |
| --- | --- | --- |
|  |  |  |

1. **In the space below, please provide comments and/or suggestions regarding your experience with the biofeedback systems, or how they can be improved. (What are the shortcomings of any of the systems? Which features would be nice to have to make your work easier? Etc.)**
